# Nodal Staging in Patients with Locally Advanced Non-Small Cell Lung Cancer: Indications, Approaches, and Impact on Downstream Treatment Selection

**DOI:** 10.3390/cancers18162561

**Published:** 2026-08-10

**Authors:** Ido Haimi, Ravi Rajaram

**Affiliations:** Department of Thoracic & Cardiovascular Surgery, The University of Texas MD Anderson Cancer Center, Houston, TX 77030, USA; ihaimi@mdanderson.org

**Keywords:** non-small cell lung cancer, mediastinal lymph node staging, endobronchial ultrasound-guided transbronchial needle aspiration, FDG-PET/CT, neoadjuvant chemoimmunotherapy, N2 disease classification, TNM ninth edition, perioperative immunotherapy, surgical resection, multidisciplinary treatment planning

## Abstract

Locally advanced non-small cell lung cancer (NSCLC) is a biologically and clinically heterogeneous disease in which mediastinal lymph node involvement remains the principal prognostic and therapeutic discriminator. Accurate nodal staging guides selection among surgical resection, definitive chemoradiotherapy, immunotherapy, and targeted therapy. This narrative review synthesizes contemporary evidence on mediastinal staging modalities, nodal disease burden, and treatment selection in stage II-III NSCLC. Contemporary staging integrates CT and FDG-PET/CT with systematic tissue confirmation using EBUS-TBNA and EUS, with surgical mediastinal staging reserved for selected high-risk or discordant cases. The ninth edition TNM N2a/N2b distinction, increasing use of neoadjuvant and perioperative immunotherapy, and molecularly guided therapy have further amplified the clinical consequences of under- or over-staging. Nodal assessment should therefore be interpreted through a multidisciplinary framework that integrates anatomy, biology, treatment response, and patient operability.

## Simple Summary

Precise mediastinal lymph node staging is fundamental to the management of locally advanced non-small cell lung cancer (NSCLC). Nodal disease status is the primary determinant of prognosis in this population and directly governs eligibility for surgery, multimodality therapy, immunotherapy, and molecularly targeted treatments. As systemic options expand and therapeutic sequencing becomes increasingly complex, accurate nodal assessment is more critical than ever. This review examines contemporary mediastinal staging approaches and illustrates how nodal evaluation drives modern treatment selection in stage II-III NSCLC.

## 1. Introduction

Lung cancer remains the leading cause of cancer-related mortality worldwide, and non-small cell lung cancer (NSCLC) accounts for approximately 85% of cases [1]. The TNM classification is the foundation for staging and treatment planning in NSCLC, with mediastinal lymph node involvement representing a critical determinant of prognosis, resectability, and multimodality treatment selection [2,3]. The distinction between N1, N2, and N3 disease determines suitability for surgery, chemoradiotherapy, immunotherapy, and molecularly targeted approaches. The ninth edition of the TNM classification further subdivides N2 disease into N2a (single-station involvement) and N2b (multi-station involvement), reflecting the prognostic heterogeneity within this category [3,4,5,6,7].

Despite major advances in immunotherapy and targeted therapy, nodal positivity continues to exert a profound influence on long-term survival and patterns of recurrence [2]. Patients with single-station N2 disease demonstrate superior outcomes compared with those with multi-station involvement, with 5-year overall survival rates differing significantly between N2a and N2b subgroups [4,7]. Furthermore, the presence of skip metastases (N2 disease without N1 involvement) confers a more favorable prognosis, with survival outcomes approaching those of N1 disease [8,9,10,11,12,13].

Risk factors for occult nodal metastasis—defined as nodal positivity in the absence of radiographic evidence of nodal disease—include central tumor location, larger tumor size (particularly >3 cm), and certain histologic subtypes. Central tumors carry a 20–25% risk of N2/N3 involvement despite normal-sized nodes on imaging, necessitating pathologic confirmation [14]. In contrast, peripheral tumors ≤ 3 cm with negative CT and PET/CT findings have a low likelihood of mediastinal involvement, making pretreatment pathologic evaluation optional in these settings [3]. In the modern era, precise nodal staging is indispensable for appropriate patient selection and optimal treatment sequencing. This review aims to synthesize current evidence on mediastinal nodal staging approaches, examine the prognostic implications of nodal disease burden, and discuss how nodal assessment informs evolving treatment paradigms in locally advanced NSCLC.

Methods. This is a narrative review. A literature search was conducted using PubMed/MEDLINE, with additional references identified from major clinical practice guidelines (NCCN, ASCO, STS, ACCP, and ERS/ESGE/ESTS), recent conference proceedings, and reference lists of key articles. The search focused on mediastinal staging modalities, nodal prognostication, and treatment selection in locally advanced NSCLC, with emphasis on publications from 2013 to 2026.

## 2. Advances in Mediastinal Nodal Staging

### 2.1. Imaging Modalities for Nodal Staging

#### 2.1.1. Computed Tomography (CT) Criteria and Limitations

**Figure 1:**
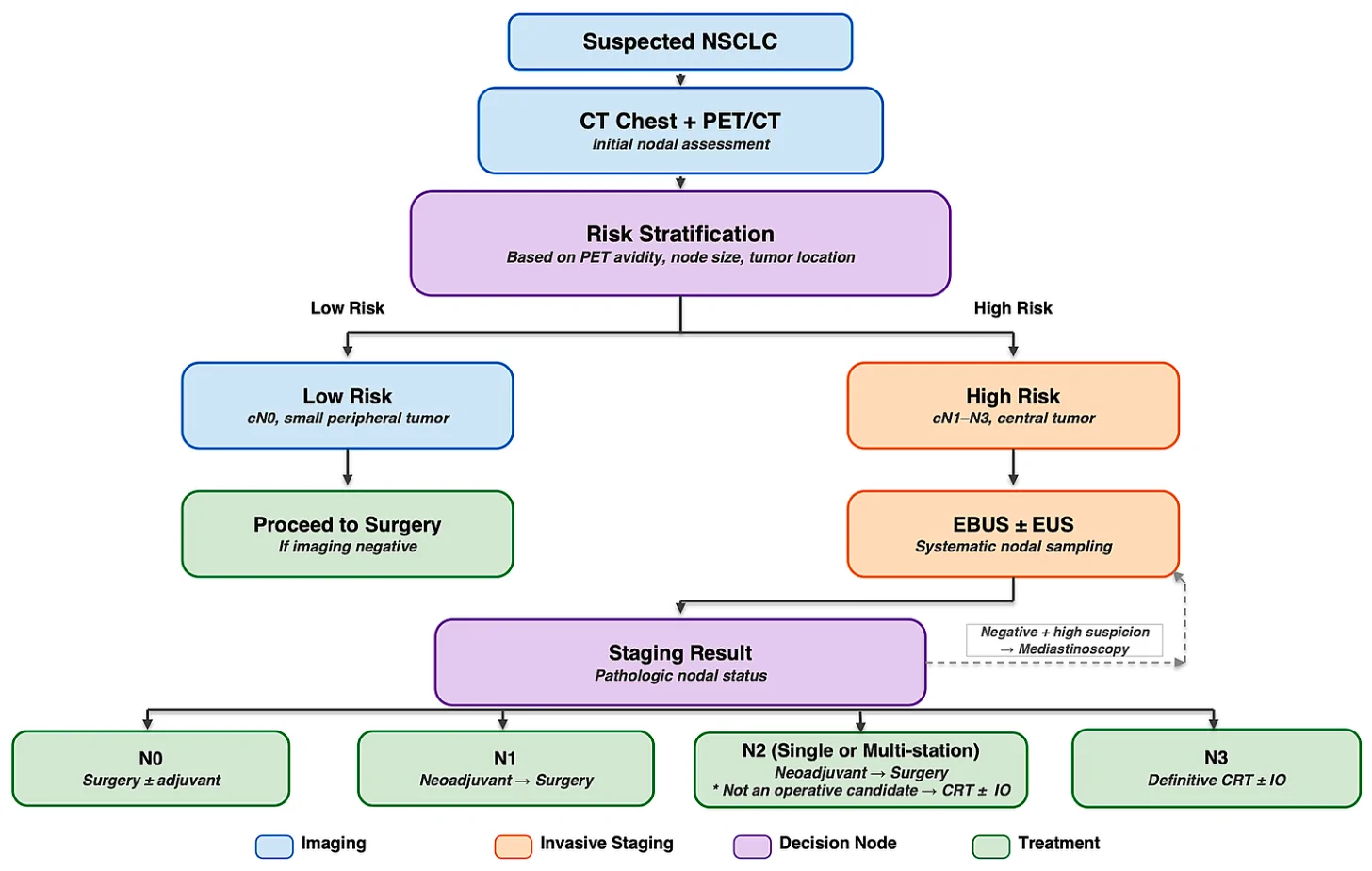
Mediastinal nodal staging workflow integrating imaging occult-risk features (central tumor, tumor > 3 cm, and suspected N1 disease), and invasive tissue confirmation.

Chest CT remains the foundational anatomic imaging modality for lung cancer staging, though its performance for mediastinal nodal assessment is limited (Figure 1). The most widely used criterion for malignant lymph node involvement is a short-axis diameter ≥ 1 cm on transverse CT imaging. However, this size-based criterion demonstrates median sensitivity of only 55% and specificity of 81% for detecting mediastinal metastases, with substantial heterogeneity across studies [14]. The limited diagnostic accuracy stems from the inability of CT to distinguish benign reactive enlargement from malignant involvement, and its failure to detect micrometastatic disease in normal-sized nodes [14].

#### 2.1.2. Positron Emission Tomography (PET)/CT Performance

**Figure 2:**
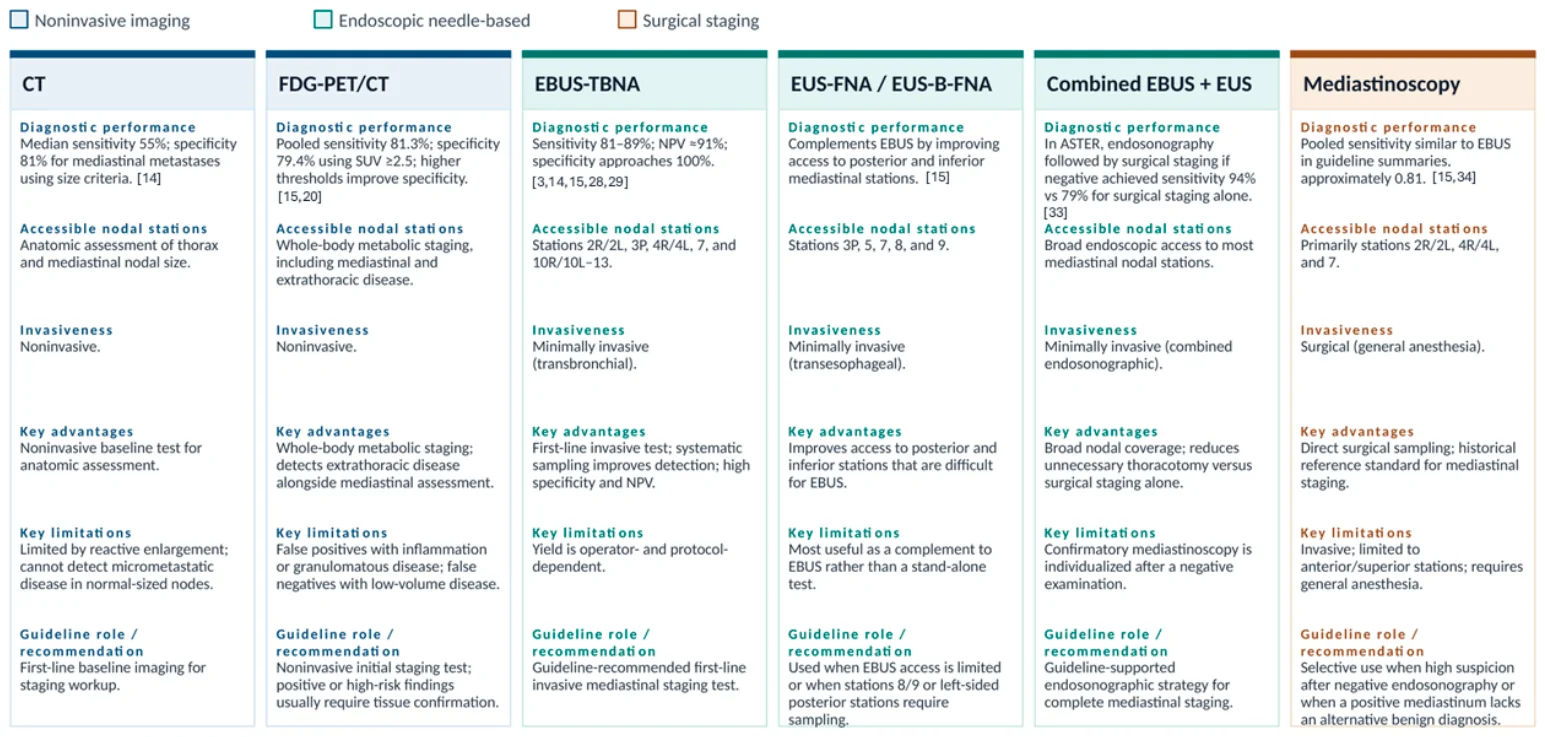
Comparative overview of mediastinal nodal staging modalities in NSCLC. Abbreviations: CT, computed tomography; FDG-PET/CT, fluorodeoxyglucose positron emission tomography/computed tomography; EBUS-TBNA, endobronchial ultrasound-guided transbronchial needle aspiration; EUS-FNA, endoscopic ultrasound-guided fine needle aspiration; NPV, negative predictive value. Values are approximate and summarized from the cited literature in the manuscript.

Fluorodeoxyglucose (FDG) PET/CT has become the standard initial staging modality, offering improved sensitivity for metabolically active nodal disease. A 2014 Cochrane Review demonstrated pooled sensitivity of 81.3% (95% CI: 70.2–88.9%) and specificity of 79.4% (95% CI: 70–86.5%) using maximum standardized uptake value (SUV) ≥ 2.5 as the criterion [15]. More recent meta-analyses confirm sensitivity ranging from 72 to 82% and specificity from 86 to 91% for mediastinal nodal staging (Figure 2) [15,16,17,18,19].

Despite these improvements over CT alone, PET/CT remains susceptible to false positives from inflammatory conditions (granulomatous disease [sarcoidosis], infections, etc.) and false negatives in micrometastatic disease, particularly in lymph nodes < 1 cm [1,15,19]. The positive predictive value varies with pretest probability, necessitating pathologic confirmation in most patients with suspected nodal involvement. Studies using higher SUV thresholds (node/mediastinal blood pool (MBP) ratio > 1.7 or node SUV > 3.9) demonstrate improved specificity (79–90%) while maintaining high sensitivity (91%) [3,15,20]. Current guideline algorithms therefore move directly to invasive mediastinal staging not only for PET/CT-positive mediastinal nodes, but also for PET-negative patients with occult N2/N3 risk factors, including central tumors, tumors > 3 cm, or radiographic/suspected hilar N1 disease [3,14]. Conversely, pretreatment invasive staging may be omitted in carefully selected peripheral tumors ≤ 3 cm with negative CT and PET/CT findings [3].

#### 2.1.3. Emerging Modalities

Total-body PET/CT demonstrates slightly higher sensitivity (86% vs. 77%) and positive predictive value (82% vs. 72%) compared to conventional PET/CT, with lower semi-quantitative thresholds for detecting positive mediastinal lymph nodes, potentially advantageous for identifying small, low FDG-avidity metastases [16]. Radiomics and artificial intelligence approaches show promise for predicting lymph node metastasis, with CT-based radiomics models achieving sensitivity of 84% and specificity of 82% (AUC 0.90), and PET/CT radiomics demonstrating sensitivity of 82% and specificity of 86% (AUC 0.90) [21]. Models incorporating both intratumoral and peritumoral radiomic features outperform those using intratumoral features alone (AUC 0.953 vs. 0.898) [22,23,24,25,26,27]. However, most radiomics and artificial intelligence models remain retrospective, single-center, and sensitive to scanner protocols, segmentation methods, and external validation. These approaches should be considered investigational adjuncts rather than substitutes for pathologic staging.

### 2.2. Invasive Staging Techniques

Endobronchial ultrasound-guided transbronchial needle aspiration (EBUS-TBNA) performance.

EBUS-TBNA has emerged as the first-line invasive mediastinal staging modality, demonstrating a pooled sensitivity of 81–89% and negative predictive value of 91% (Figure 2). The procedure provides access to nodal stations 2R/2L, 3P, 4R/4L, 7, and 10R/10L-13, with specificity approaching 100% and minimal procedure-related morbidity [3,14,15,28,29].

Systematic versus targeted EBUS approaches have been compared, with systematic sampling of all visible lymph nodes ≥ 5 mm demonstrating superior detection of occult disease. In the SEISMIC trial, systematic EBUS-TBNA identified PET-occult mediastinal nodal metastases in 12% of patients with locally advanced NSCLC, altering treatment decisions in all cases and necessitating extended radiation target volumes in two-thirds. A separate study found systematic EBUS detected PET-occult disease in 33% of patients considered for definitive chemoradiation [30,31]. These data also underscore that EBUS quality depends on systematic nodal assessment, operator experience, and institutional protocols; diagnostic yield may vary substantially across centers [14,28,29,32].

#### 2.2.1. Combined EBUS and EUS

Adding EUS to EBUS allows access to more lymph node stations (3P, 5, 7, 8, and 9) and improves detection of hidden N2/N3 disease, reducing the number of patients needed to stage from about 14 to 7. However, a small trial showed no added benefit when EBUS was done first [15].

#### 2.2.2. EBUS Versus Mediastinoscopy

The ASTER trial demonstrated that endosonography (EBUS + EUS) followed by surgical staging if negative had higher sensitivity (94% vs. 79%, *p* = 0.02) and fewer unnecessary thoracotomies (7% vs. 18%, *p* = 0.02) compared with surgical staging alone, while being more cost-effective (Figure 2). Pooled estimates show equivalent sensitivity between EBUS (0.81) and mediastinoscopy (0.81) for diagnosing mediastinal lymph node involvement [14,15,33,34]. The 2023 randomized ASTER-2 trial found that confirmatory mediastinoscopy after negative systematic endosonography can be omitted, with unforeseen N2 rates of 8.8% after immediate resection being noninferior to 7.7% after mediastinoscopy [35].

Guideline recommendations are broadly aligned in supporting endosonography as the first invasive staging modality, but they differ in the threshold for mandatory tissue confirmation. The ACCP recommends invasive staging for an intermediate suspicion of N2/N3 involvement, including central tumors or N1 lymph node enlargement despite a radiographically normal mediastinum, and supports needle-based endoscopic staging as the preferred initial test [14]. NCCN considers pretreatment pathologic mediastinal evaluation optional only for peripheral tumors ≤ 3 cm with negative CT and PET/CT, while recommending invasive mediastinal staging for central tumors and other higher-risk clinical stage I-II presentations planned for resection [3]. ASCO reports similar pooled sensitivity for EBUS and mediastinoscopy and supports endoscopic staging first, with confirmatory mediastinoscopy reserved for selected patients in whom clinical suspicion remains high after a negative needle technique [15]. The 2026 ERS/ESGE/ESTS guideline similarly recommends endosonography over mediastinoscopy for mediastinal nodal tissue staging, supports systematic rather than targeted staging as the minimum standard, and does not recommend routine add-on mediastinoscopy after negative endosonography (Figure 2) [36].

Important caveat: When EBUS-TBNA is negative for malignancy despite a clinically positive mediastinum on PET/CT and/or CT, the result should be reviewed in a multidisciplinary setting and further investigation, including mediastinoscopy in selected patients, should be considered before surgical resection to minimize false-negative staging. This is particularly relevant when bronchoscopic biopsies do not demonstrate an alternative histologic diagnosis (e.g., granulomatous inflammation).

Additionally, recent data suggest systematic EBUS-TBNA diagnoses a larger extent of mediastinal disease in 9% of cases compared with targeted approaches, identifying N3 disease in patients with N2 on PET/CT and N2b in those with N2a on imaging [3,32].

### 2.3. Mediastinal Re-Staging and Novel Approaches

#### Approaches to Restage After Induction Therapy

Pathologic downstaging or mediastinal clearance (i.e., ypN0-1) has been associated with improved OS and PFS [2]. However, phase III randomized clinical trials studying multimodality therapy, including surgery, in patients with NSCLC and N2 disease have not mandated invasive mediastinal re-staging in their protocols and only excluded patients in the event of disease progression after neoadjuvant therapy [2]. Consequently, re-staging the mediastinum following neoadjuvant therapy is not typically performed as a matter of routine. Re-staging may be considered based on specific patient considerations, such as a high-risk surgical case where an excellent response to neoadjuvant treatment is required to justify expected perioperative morbidity. That said, for patients with resectable NSCLC and persistent N2 disease after induction therapy, but without progression, proceeding with surgical resection is generally appropriate [2].

Radiographic re-staging after induction therapy is difficult to interpret, but CT ± FDG-PET/CT should be performed to exclude disease progression, rule out immunotherapy-related pneumonitis, and for surgical planning [3].

## 3. Prognostic Significance of Nodal Involvement

### 3.1. Nodal Status and Survival Outcomes

**Figure 3:**
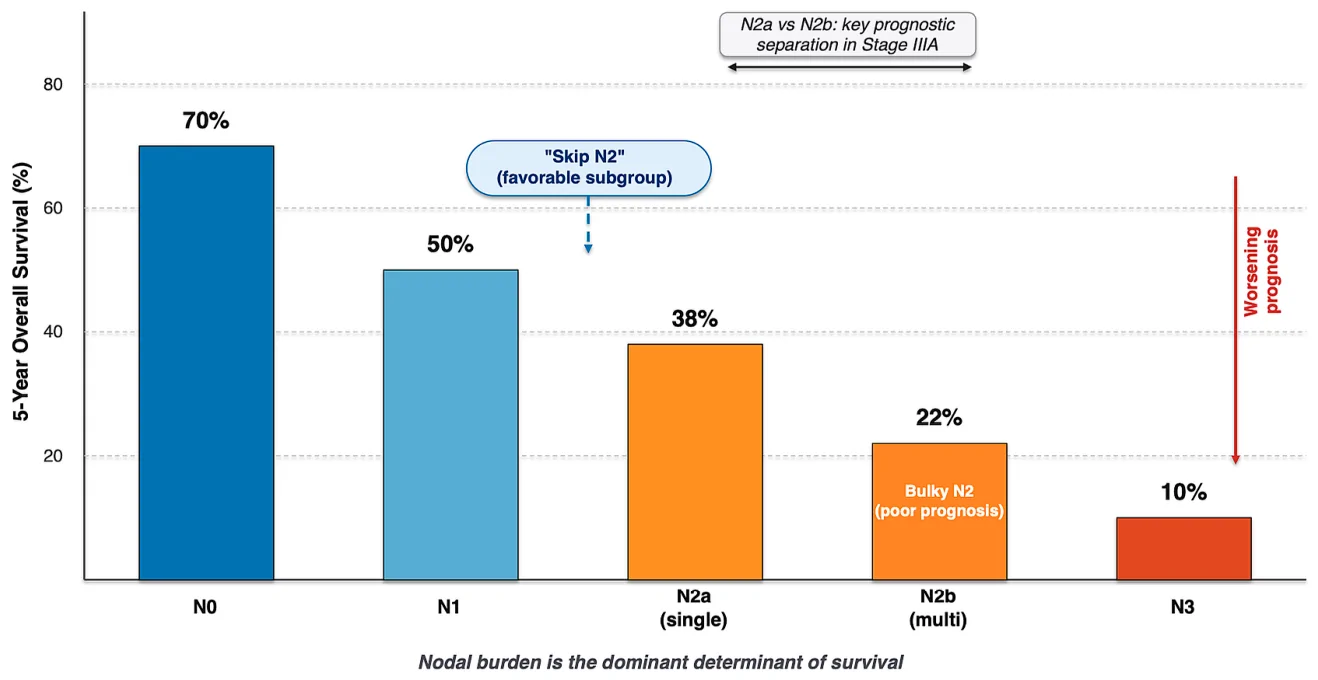
Schematic representation of the prognostic gradient by nodal status. Survival estimates are approximated from published IASLC ninth edition TNM validation studies and related large datasets [4,5,7]; the figure illustrates the general trend and does not represent a single Kaplan–Meier analysis.

Survival outcomes in non-small cell lung cancer decline in a stepwise fashion with increasing nodal burden (Figure 3) [2]. The ninth edition TNM classification recognizes this heterogeneity by subdividing N2 disease into N2a (single-station) and N2b (multi-station), with validation studies demonstrating significant prognostic differences [3,4,5,6,7]. Patients with N3 involvement have particularly poor outcomes, with 5-year survival rates typically at 10% [2].

### 3.2. Extent of Nodal Metastases

Beyond the categorical N descriptor, several nodal characteristics independently influence prognosis, including the number of involved lymph nodes, the number of nodal stations, extranodal extension, and the presence of skip metastases [2,7]. Patients with single-station, non-bulky N2 disease consistently demonstrate superior outcomes compared with those with multi-station involvement, supporting a more granular interpretation of nodal stage [2,7]. These observations have informed refinements to the TNM classification and underpin contemporary risk-adapted treatment strategies.

Bulky mediastinal lymphadenopathy is defined as lymph nodes ≥ 2.5 cm in short-axis diameter on CT imaging or those showing extranodal involvement or groupings of multiple smaller lymph nodes [2]. Retrospective series have shown that patients with NSCLC and bulky N2 disease experience worse long-term cancer-specific outcomes compared with patients with non-bulky lymph nodes [2].

### 3.3. Location and Pattern of Metastases

Skip metastasis are defined as those with N2 disease without N1 (skip N2). Multiple studies have demonstrated that patients with skip N2 metastases have significantly improved survival compared to those with sequential N1-N2 involvement, with some series showing 5-year survival rates approaching those of N1 disease [8,9,10,11,12,13].

### 3.4. Nodal Burden and Nodal Response

Pathologic complete response (pCR) and nodal downstaging after neoadjuvant therapy are powerful prognostic indicators. In the CheckMate 816 trial, patients with a pCR after neoadjuvant nivolumab plus chemotherapy had a 5-year overall survival of 95.3% compared with 55.7% among those without such a response (HR for death, 0.11; 95% CI, 0.04 to 0.36) [37]. Notably, no deaths from lung cancer occurred in patients who had a pCR [37].

Achieving ypN0 status post-therapy strongly predicts favorable long-term survival. In a National Cancer Database analysis of patients receiving neoadjuvant chemoimmunotherapy for resectable NSCLC, 5-year OS was 84% for ypN0, 64% for ypN1, and 51% for ypN2 (*p* < 0.001) [38]. Patients achieving ypN0 status had similar outcomes regardless of pretreatment cN1 or cN2 status, while pretreatment cN stage became less prognostically relevant after nodal downstaging [38].

The combination of tumor and nodal responses is significantly associated with prognosis. Patients who achieved both ypT0 and ypN0 (pCR; combined good responders) demonstrated the most favorable event-free survival outcomes [39]. A meta-analysis demonstrated that achieving pCR or MPR was associated with improved event-free survival (EFS) in the intention-to-treat population (pCR, HR = 0.13; MPR, HR = 0.18), with 24-month EFS rates of 94% and 88% for patients who achieved pCR and MPR, respectively [40].

Although pCR, MPR, and nodal clearance are clinically useful, they remain imperfect surrogate endpoints. pCR and MPR are strongly associated with EFS, but available data linking these endpoints to OS are less mature and may be confounded by adjuvant therapy and trial-specific treatment sequences [37,40]. In immunotherapy-treated tumors, histologic regression may include immune-mediated stromal remodeling, fibrosis, and nodal immune activation; therefore, pCR should be interpreted together with nodal response, radiographic findings, and long-term follow-up rather than as a standalone determinant of cure.

## 4. Nodal Staging and Treatment Selection

### 4.1. Nodal Status and Treatment Selection

**Figure 4:**
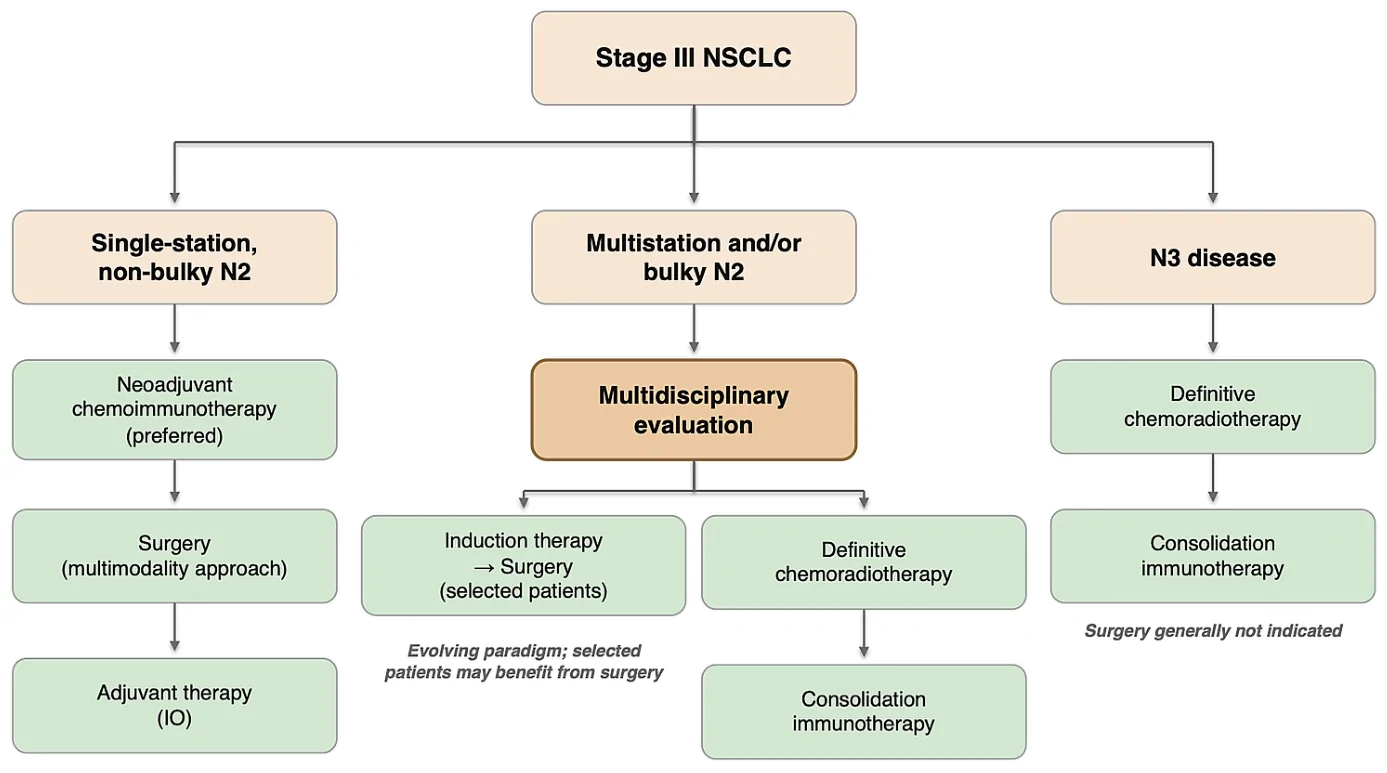
Treatment algorithm stratified by nodal disease burden in stage III NSCLC, including the two principal pathways for discrete resectable N2 disease: induction chemoimmunotherapy followed by surgery versus definitive chemoradiotherapy followed by consolidation immunotherapy. Treatment framework stratified by nodal disease burden in stage III NSCLC (paradigm in patients without EGFR/ALK mutations). While single-station non-bulky N2 is commonly managed with multimodality therapy including surgery, the role of surgery in multi-station or bulky N2 disease is evolving and should be individualized. N3 disease is generally treated with definitive chemoradiotherapy.

Nodal status remains the central determinant of therapeutic strategy in locally advanced non-small cell lung cancer, but its role is evolving in the era of immunotherapy and multimodality treatment (Figure 4) [2,3]. Stage III NSCLC is highly heterogeneous, and the choice and sequence of multimodality treatment vary substantially across clinicians and institutions [15].

#### 4.1.1. N0/N1 (Stage I–II)

Upfront surgery remains standard for N0 patients with tumors < 4 cm in size (clinical stage I disease). Because recent phase III trials of neoadjuvant chemoimmunotherapy included N1 disease, induction therapy is increasingly favored in this group [2,3]. Accurate staging is essential to avoid undertreatment.

#### 4.1.2. N2 Disease (Stage IIIA)

N2 disease represents a biologically heterogeneous spectrum where treatment decisions should be individualized rather than strictly algorithmic (Figure 4).

- Single-station, non-bulky N2: Multimodality therapy with induction chemoimmunotherapy followed by surgical resection remains an accepted approach for medically operable patients after multidisciplinary review [2,3]. Definitive concurrent chemoradiotherapy followed by consolidation immunotherapy remains an alternative pathway when resectability, operative risk, response, or patient preference favor nonoperative treatment [15,41,42,43,44].
- Bulky N2: The STS consensus defines bulky mediastinal lymphadenopathy as lymph nodes ≥ 2.5 cm in short-axis diameter on CT imaging, extranodal involvement, or groupings of multiple smaller nodes, whereas the NCCN institutional survey uses ≥3 cm as an operational threshold [2,3]. This variability should be recognized when interpreting recommendations. Bulky N2 disease is an adverse feature and is most often managed with definitive chemoradiotherapy followed by consolidation immunotherapy, although select patients may still be considered for surgery in a multimodality context [2,3].
- Multi-station N2: The role of surgery remains controversial. Contemporary neoadjuvant chemoimmunotherapy trials included selected patients with multi-station N2 disease and demonstrated meaningful pathologic response rates; exploratory analyses from CheckMate 77T showed comparable pCR rates in multi-station N2 versus the broader N2 cohort (37.5% vs. 24.4%) [45]. However, this evidence is based primarily on pathologic response rather than mature subgroup-specific OS data. The STS consensus notes that patients with multi-station N2 disease have historically not been considered candidates for surgical resection due to poor long-term outcomes, though present-day systemic regimens may extend indications for resection as long-term survival data become available [2,15]. Surgery may be considered only in highly selected patients with non-bulky, limited multi-station disease after multidisciplinary review; enrollment in clinical trials is strongly encouraged.

For discrete resectable N2 disease, the treatment algorithm should be interpreted as two principal pathways: induction chemoimmunotherapy followed by surgery with consideration of adjuvant systemic therapy, or definitive concurrent chemoradiotherapy followed by consolidation immunotherapy or molecularly guided consolidation where appropriate [3,15,41]. No contemporary randomized trial has directly compared these strategies in resectable N2 disease, making multidisciplinary patient selection essential.

#### 4.1.3. N3 Disease (Stage IIIB)

Contralateral mediastinal and/or any supraclavicular nodal involvement is generally considered unresectable and treated with definitive chemoradiotherapy [2,3]. Pathologic confirmation is recommended prior to excluding surgical options.

### 4.2. Radiation and Nodal Coverage

In definitive chemoradiotherapy, nodal staging directly determines radiation field design. PET-positive and biopsy-proven nodal stations are included in the high-dose volume, while elective nodal irradiation is typically omitted to reduce toxicity [3].

Systematic invasive staging improves target delineation. The SEISMIC trial demonstrated that routine EBUS identified PET-occult nodal disease in 12% of patients, resulting in modification of radiation fields [30].

Involved-field irradiation is associated with low rates of isolated nodal relapse and may permit dose escalation compared with elective nodal irradiation [3].

### 4.3. Adjuvant Therapy Decisions

#### 4.3.1. Postoperative Radiation Therapy (PORT)

In completely resected N2 disease, randomized trials (Lung ART and PORT-C) showed improved locoregional control but no overall survival benefit [2,3,15,46]. PORT may be considered in selected high-risk patients, including those with multi-station involvement, extracapsular extension, or inadequate nodal assessment [3].

#### 4.3.2. Adjuvant Immunotherapy

Adjuvant immunotherapy is now commonly incorporated for resected stage II–III disease, with utilization varying by PD-L1 status [3].

### 4.4. Special Situations

#### 4.4.1. Indeterminate Nodal Disease

When biopsy is not feasible, management includes empiric treatment based on clinical suspicion or surgical staging, including minimally invasive approaches or anterior mediastinotomy for select stations [particularly for stations 5 and 6] [2,3].

#### 4.4.2. Occult N2 Discovered Intraoperatively

Unexpected N2 disease is typically managed with completion of resection followed by adjuvant therapy, although bulky disease may prompt reassessment of the operative plan if early in the case. This scenario underscores the importance of thorough preoperative staging [3].

## 5. Evolving Therapeutic Paradigms: Personalized Approaches

### 5.1. Integration of Immunotherapy in Stage III Patients

The PACIFIC trial established consolidation durvalumab after definitive chemoradiotherapy as standard of care for unresectable stage III NSCLC, with durable survival benefit [15,41,42,43,44]:

- 5-year OS: 42.9% vs. 33.4% (HR 0.72; 95% CI, 0.59–0.89).
- 5-year PFS: 33.1% vs. 19.0% (HR 0.55; 95% CI, 0.45–0.68).
- Median OS: 47.5 vs. 29.1 months (HR 0.72; 95% CI, 0.59–0.89).

Benefit was consistent across nodal subgroups. PACIFIC-5 further confirmed improved PFS after either concurrent or sequential chemoradiotherapy (14.0 vs. 6.5 months; HR 0.75; *p* = 0.038) [41].

#### Concurrent Immunotherapy with Chemoradiotherapy

PACIFIC-2 did not show benefit for concurrent durvalumab with chemoradiotherapy (PFS HR 0.85; 95% CI, 0.65–1.12; *p* = 0.247; OS HR 1.03; 95% CI, 0.78–1.39; *p* = 0.823) [47]. Consolidation durvalumab remains standard and is now extended to unresectable stage II disease [3,47].

### 5.2. Phase III Neoadjuvant and Adjuvant Immunotherapy Trials

#### 5.2.1. CheckMate 816

Neoadjuvant nivolumab plus chemotherapy improved outcomes versus chemotherapy alone [37,48,49,50,51]:

- 5-year OS: 65.4% vs. 55.0% (HR 0.72; 95% CI, 0.52–1.00; *p* = 0.048).
- pCR: 24.0% vs. 2.2% (OR 13.94; 99% CI, 3.49–55.75; *p* < 0.001).
- Median EFS: 31.6 vs. 20.8 months (HR 0.63; 97.38% CI, 0.43–0.91; *p* = 0.005).

Benefit was greatest in stage IIIA and PD-L1 ≥ 1% tumors, though FDA approval of this regimen was granted to all patients with stage II-III disease [50,51].

#### 5.2.2. Perioperative Immunotherapy

Phase III trials (CheckMate 77T, KEYNOTE-671, AEGEAN, Neotorch, RATIONALE-315) consistently demonstrate improved EFS, pCR, and emerging OS benefit with perioperative checkpoint inhibition [45,52,53,54]. This establishes perioperative immunotherapy as a new standard for resectable stage II–III disease.

#### 5.2.3. Adjuvant Immunotherapy

IMpower010 showed DFS and OS benefit with adjuvant atezolizumab in PD-L1–positive stage II–IIIA disease [55,56,57,58].

PEARLS/KEYNOTE-091 demonstrated improved DFS in this population with adjuvant pembrolizumab (HR 0.76) [57].

### 5.3. Targeted Therapies in Resectable Lung Cancer

#### 5.3.1. ADAURA Trial

Adjuvant osimertinib (80 mg daily for 3 years) in resected EGFR-mutant (ex19del/L858R) stage IB–IIIA NSCLC significantly improved DFS and OS versus placebo, with or without prior adjuvant chemotherapy [56,57]:

- DFS (stage II–IIIA): HR 0.23 (95% CI, 0.18–0.30); 4-year DFS 70% vs. 29%.
- DFS (stage IB–IIIA): HR 0.27 (95% CI, 0.21–0.34); 4-year DFS 73% vs. 38%.
- OS (stage II–IIIA): HR 0.49 (95.03% CI, 0.33–0.73; *p* < 0.001); 5-year OS 85% vs. 73%.
- OS (stage IB–IIIA): HR 0.49 (95.03% CI, 0.34–0.70; *p* < 0.001); 5-year OS 88% vs. 78%.
- CNS DFS (stage II–IIIA): HR 0.24 (95% CI, 0.14–0.42).

#### 5.3.2. ALK-Positive Disease

Adjuvant alectinib (600 mg twice daily for 2 years) versus platinum-based chemotherapy in resected ALK-positive stage IB (≥4 cm)–IIIA NSCLC demonstrated a 76% reduction in risk of recurrence or death [3,59]:

- DFS (stage II–IIIA): HR 0.24 (95% CI, 0.13–0.45; *p* < 0.0001); 2-year DFS 93.8% vs. 63.0%.
- DFS (ITT, stage IB–IIIA): HR 0.24 (95% CI, 0.13–0.43; *p* < 0.0001); 2-year DFS 93.6% vs. 63.7%.
- CNS DFS: HR 0.22 (95% CI, 0.08–0.58); brain recurrence in 3.1% vs. 11.0%.
- OS data remain immature (2.3% event rate at data cutoff).

#### 5.3.3. Molecular Testing

All resectable stage II–IIIA patients require biomarker testing (EGFR, ALK, PD-L1) prior to treatment selection [3,57,60]. This is critical given limited immunotherapy benefit and potential toxicity in EGFR-mutant or ALK fusion patients [57].

### 5.4. Biomarkers and Future Strategies

Circulating tumor DNA (ctDNA) is an emerging biomarker for minimal residual disease (MRD) with strong prognostic value. Post-treatment positivity predicts recurrence with high specificity [61,62,63,64,65,66,67,68,69,70,71,72].

In CheckMate 816, ctDNA clearance correlated with improved OS (75.0% vs. 52.6%) [37]. MRD status may eventually guide adjuvant therapy selection, though current limitations include assay variability, cost, turnaround time, and uncertain sensitivity in low-volume disease [61,62,67,68,69,70]. Trials such as MERMAID are evaluating ctDNA-guided treatment strategies [61,62]. As a potential future “liquid nodal biopsy,” ctDNA could complement anatomic and pathologic staging by providing a noninvasive signal of tumor burden, nodal involvement, and treatment response [68]. However, ctDNA is not currently validated for mediastinal staging in stage I-III disease. Low shedding in early or low-volume tumors, platform variability, and the absence of prospective staging-directed trials preclude using ctDNA to replace EBUS, EUS, or surgical staging at present [61,67,68].

#### Tumor Mutational Burden (TMB), PD-L1, and Tumor Microenvironment

Higher TMB and PD-L1 correlate with response to neoadjuvant therapy, though TMB is not yet standard [73]. Treatment heterogeneity likely reflects tumor-intrinsic and microenvironmental factors in addition to anatomic nodal stage, including immune-cell infiltration, stromal remodeling, and tertiary lymphoid structures. These biomarkers may eventually complement nodal staging but remain investigational. Recent multiomic data suggest that node-dominant T1N2 tumors may exhibit immune-active “hot” phenotypes, whereas tumor-dominant T4N0 tumors may show stromal remodeling, immune exclusion, and immunosuppression [74]. These translational findings are preliminary and should be considered hypothesis-generating.

## 6. Conclusions and Future Directions

Accurate mediastinal nodal staging in locally advanced NSCLC remains central to treatment selection and outcomes. The novelty of this review is its integration of contemporary staging evidence with the rapidly evolving immunotherapy and targeted therapy landscape, particularly in the context of the ninth edition TNM classification. Nodal status still dictates the use of surgery, chemotherapy, radiotherapy, immunotherapy, and targeted agents, but it must now be interpreted alongside tumor biology, molecular status, response to induction therapy, and patient operability.

Several limitations of the current evidence should temper interpretation. EBUS performance is operator and protocol dependent; AI and radiomics studies are frequently retrospective, single-center, and incompletely validated; and several perioperative immunotherapy trials still require longer follow-up to clarify the relationship between pCR, MPR, nodal downstaging, EFS, and OS. The role of surgery in multi-station N2 disease remains especially unresolved, with current enthusiasm driven largely by pathologic response rather than mature survival data in this subgroup. As a narrative review, this article is also subject to literature-selection bias and does not provide the reproducibility of a systematic review.

Future work should define which patients with N2 disease benefit most from surgery after induction chemoimmunotherapy, compare surgical and nonsurgical multimodality pathways in the modern immunotherapy era, and clarify whether adjuvant immunotherapy adds benefit after neoadjuvant chemoimmunotherapy in patients achieving pCR. Further gains will likely come from enhanced imaging, standardized endoscopic staging, validated artificial intelligence-driven predictive models, tumor microenvironment-based risk stratification, and ctDNA-based MRD assessment. ctDNA as a “liquid nodal biopsy” remains a promising but speculative future direction rather than a current substitute for pathologic mediastinal staging.

## Data Availability

No new data were created or analyzed in this study. Data sharing is not applicable.
